# Glucose-Induced Expression of DAPIT in Pancreatic β-Cells

**DOI:** 10.3390/biom10071026

**Published:** 2020-07-10

**Authors:** Alberto Leguina-Ruzzi, Anežka Vodičková, Blanka Holendová, Vojtěch Pavluch, Jan Tauber, Hana Engstová, Andrea Dlasková, Petr Ježek

**Affiliations:** Department of Mitochondrial Physiology, No.75, Institute of Physiology of the Czech Academy of Sciences, 142 20 Prague, Czech Republic; AlbertoAndres.LeguinaRuzzi@fgu.cas.cz (A.L.-R.); anezka.kahancova@fgu.cas.cz (A.V.); blanka.holendova@fgu.cas.cz (B.H.); vojtech.pavluch@fgu.cas.cz (V.P.); jan.tauber@fgu.cas.cz (J.T.); hana.engstova@fgu.cas.cz (H.E.); andrea.dlaskova@fgu.cas.cz (A.D.)

**Keywords:** mitochondria, USMG5/DAPIT, glucose-stimulated insulin secretion, glucose-induced expression, membrane subunits of ATP synthase, ATP synthase oligomers mitochondrial cristae morphology

## Abstract

Transcript levels for selected ATP synthase membrane F_O_-subunits—including DAPIT—in INS-1E cells were found to be sensitive to lowering glucose down from 11 mM, in which these cells are routinely cultured. Depending on conditions, the diminished mRNA levels recovered when glucose was restored to 11 mM; or were elevated during further 120 min incubations with 20-mM glucose. Asking whether DAPIT expression may be elevated by hyperglycemia in vivo, we studied mice with hyaluronic acid implants delivering glucose for up to 14 days. Such continuous two-week glucose stimulations in mice increased DAPIT mRNA by >5-fold in isolated pancreatic islets (ATP synthase F_1_α mRNA by 1.5-fold). In INS-1E cells, the glucose-induced ATP increment vanished with DAPIT silencing (6% of ATP rise), likewise a portion of the mtDNA-copy number increment. With 20 and 11-mM glucose the phosphorylating/non-phosphorylating respiration rate ratio diminished to ~70% and 96%, respectively, upon DAPIT silencing, whereas net GSIS rates accounted for 80% and 90% in USMG5/DAPIT-deficient cells. Consequently, the sufficient DAPIT expression and complete ATP synthase assembly is required for maximum ATP synthesis and mitochondrial biogenesis, but not for insulin secretion as such. Elevated DAPIT expression at high glucose further increases the ATP synthesis efficiency.

## 1. Introduction

Pancreatic β-cells sense glucose and respond to its elevated concentration in rich capillaries of the pancreatic islets by exocytosis of insulin granules [1,2,3,4,5,6,7]. The increased mitochondrial ATP synthesis due to the elevated oxidative phosphorylation (OXPHOS) represented a consensual canonical mechanism of glucose-stimulated insulin secretion (GSIS). We recently demonstrated that also NADPH-oxidase-4- (NOX4-) mediated H_2_O_2_ release is fundamentally required for GSIS in addition to ATP [8]. The elevated OXPHOS is transformed to the increased ATP/ADP ratio at the peri-plasma membrane loci proximal to the ATP-sensitive K^+^ channel. The channel closes when both conditions are fulfilled, i.e., elevated ATP and elevated H_2_O_2_ [8], which results in the depolarization of the plasma membrane. This activates voltage-gated L-type Ca^2+^ channels (Ca_L_). The subsequent Ca^2+^ entry to the cytosol initiates the insulin exocytosis of insulin granules.

To match physiological postprandial glucose concentrations with the sensitivity range of the glucose sensor, numerous factors delicately tune glucose-concentration dependence for the insulin release mechanism. The insulin-independent glucose transporter GLUT-2 (GLUT-1 in humans) rapidly equilibrates blood glucose with the cytosolic glucose in β-cells [9]. The glucokinase (hexokinase IV) is insensitive to the inhibition by glucose-6-phosphate [10]. Moreover, low activities of lactate dehydrogenase and pyruvate dehydrogenase kinase enable a nearly 100% utilization of pyruvate by OXPHOS [1,2,3,4,5,6].

We found that also the inhibitory factor IF1 of the mitochondrial ATP synthase belongs to the key proteins, ensuring the physiological range of the glucose sensor [11,12]. The IF1 slightly inhibits synthesis of ATP, thus setting the range for elevation of phosphorylating respiration and insulin release above 3-mM glucose [11,13], with half-activation between 3.5 and 4-mM and saturation above 8-mM glucose [11,12]. When such a slight in vivo inhibition was largely cancelled using the silencing of IF1, the elevation of respiration and OXPHOS occurred at very low glucose concentration approaching to zero [9]. Simultaneously, the half-activation for the insulin release dose–response was shifted to the range of 0–2-mM glucose in INS-1E cells [9]. In contrast, overexpression of IF1 substantially blocked GSIS [13].

Another peculiar aspect of the pancreatic β-cell glucose sensor is the steepness of the related dose–response, i.e., insulin release dependence vs. glucose concentration [11,12,14,15,16]. The revealed narrowing of mitochondrial cristae may hypothetically contribute to this steepness, such as observed in INS-1E cells during GSIS [17] or upon the increased load of cell-permeant Krebs cycle substrate [18,19]. Moreover, the ATP synthase dimers, tetramers [20] or speculatively higher oligomers are associated within the rows along the crista rims [21,22,23,24,25,26].

**Figure 1:**
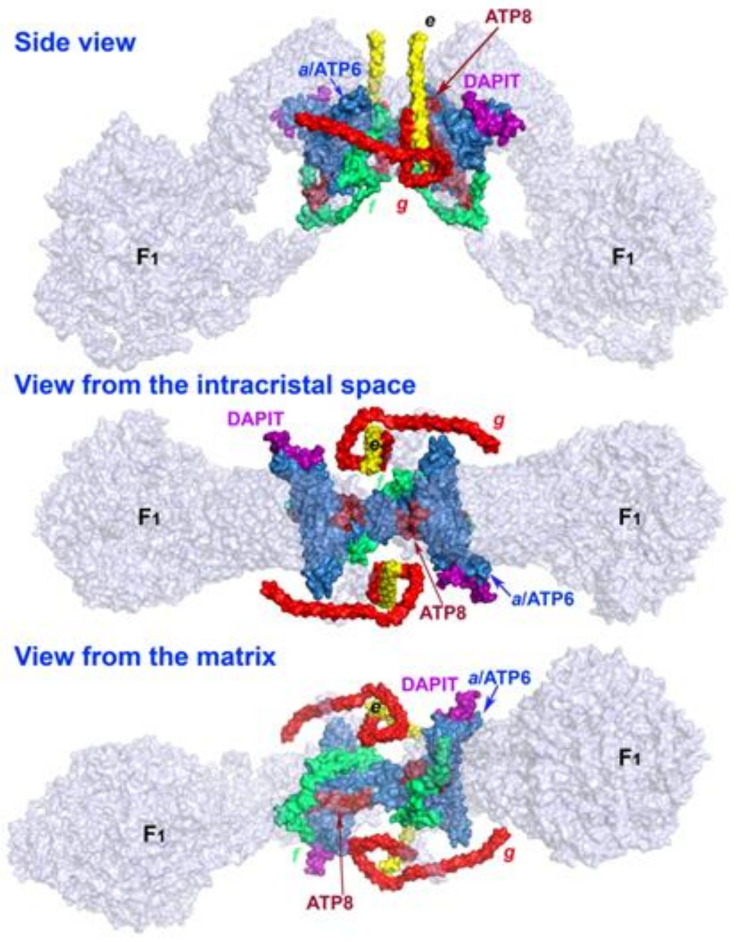
DAPIT location within the ATP synthase structure. Neighbor subunits of DAPIT are shown within the structure of membrane F_O_-sector of the ATP synthase. DAPIT and the surrounding subunits are depicted in color, the remaining subunits are transparent. A side view represents a transversal section of the crista rim; a “bottom” view is from the intracristal space directly viewing the inner sharpest IMM bend, when one considers the F_1_ moiety being positioned at the top. A top view is also the top view for the crista rim and the top of the sharp edge of the IMM bent. The ATP synthase structure was derived from the atomic model for the dimeric F_O_ region of mitochondrial ATP synthase published by Guo H. et al. [37], pdb code 6b8 h. The structure was visualized using the PyMOL Molecular Graphics System, Version 1.8 Schrödinger, LLC.

The stabilization within the rims may depend on the presence and correct assembly of the membrane embedded subunits ***e***, ***f***, ***g*** of the membrane F_O_-sector [20,26]; or DAPIT, which is the peripheral outmost subunit of the ATP synthase (Figure 1), added lastly upon the assembly [26]. DAPIT is a product of the rat *Usmg*5 gene [27,28,29,30,31]. Similarly, Mic10 was suggested to be inserted between the neighbor dimers or tetramers of the ATP synthase [32,33]. Likewise, crosslinking of two F_1_ moieties from the neighbor ATP synthase dimers by the un-phosphorylated dimeric inhibitory factor IF1 can stabilize the rim [20], but at the expense of ATP synthesis, since this interaction is inhibitory [13]. The inner mitochondrial membrane (IMM) bending itself may be ensured by subunits ***e*** and ***g*** [20]. In porcine ATP synthase a self-standing subunit ***k*** belongs to those outmost subunits [14], whereas a yeast subunit ***k*** was originally annotated as a DAPIT ortholog [26].

DAPIT is bound to the (mtDNA-) mitochondria-encoded subunit ***a*** (Figure 1). DAPIT may also interact with the second mtDNA-encoded subunit ATP8 [20,26]. However, a tetramer formation may be facilitated by an interaction of DAPIT with the subunit ***g*** within an interface between all four monomers [20]. Such interface was termed site three among altogether six sites determining the tetramer structure. Since DAPIT was found to be assembled as the last subunit into the F_O_-sector structure [26], we may speculate that its stoichiometry to the F_O_ moiety may be variable.

Moreover, a fraction of Mic10 was found to be associated with the ATP synthase and hypothetically can crosslink the neighbor dimers [32,33]. Mic10 otherwise ensures 90° curvature of the IMM at the crista outlets being a part of the MICOS complex [32,33]. Besides Mic10 [32,33], also DAPIT has been suggested to crosslink rows of ATP synthase dimers at crista rims and thus stabilize them [26].

The DAPIT was originally termed diabetes-associated protein in insulin-sensitive tissue. At a complete stoichiometry, the physiologically established dimers and tetramers of the ATP synthase possess two and four F_O_-sectors, respectively, each containing a single DAPIT subunit, embedded into IMM, actually spanning from its intracristal surface to the matrix IMM surface (Figure 1). DAPIT-knockdown in He–La cells reportedly led to a slightly decreased ATP synthesis activity and slower cell growth, while transcripts for the F_1_-sector subunits α and β did not change [29]. Moreover, a homozygous splice-site mutation (c.87 + 1G > C) in *Dapit*/*Usmg5* gene reportedly determines suppression of ATP synthase dimers and inhibition of ATP synthesis in patient’s fibroblasts [34]. This particular DAPIT mutation causes altered mitochondrial cristae in fibroblasts of Leigh syndrome patients [35]. Possibly similar or related mechanisms as caused by the DAPIT mutation are involved in sensitizing of pancreatic cancer cells to inhibitors impairing their survival [36].

Despite its name pointing to diabetes (alternatively to *upregulated in skeletal muscle gene 5*, *Usmg5* in the rat genome), the DAPIT protein has not been studied in pancreatic β-cells and there is no knowledge concerning its effect on the regulation of the ATP synthesis efficiency and its role in GSIS. Consequently, we attempted to study possible DAPIT role, using the rat pancreatic β-cell line INS-1E.

## 2. Materials and Methods

### 2.1. Model Cells

#### 2.1.1. Cell Culturing

Rat insulinoma INS-1E cells were kindly provided by Prof. Maechler, University of Geneva or purchased from AddexBio (San Diego, CA, USA; cat. No. C0018009). The results were invariant of the source as documented in previous reports [8,11,12,38,39]. Cells were cultivated with 11-mM glucose in RPMI 1640 medium with l-glutamine, supplemented with 10-mM HEPES, 1-mM pyruvate, 5% (*v/v*) fetal calf serum, 50-μmol/L mercaptoethanol, 50 IU/mL penicillin and 50-μg/mL streptomycin [38,39]. Prior to each experiment, cells were preincubated for 30 min (two washes of 15 min each) in cultivation medium containing 3-mM glucose and 2.5% of fetal calf serum.

#### 2.1.2. Silencing

Silencing of *Usmg*5/*Dapit* expression was performed with two database-registered siRNAs (#HA13041841, #HA13041842 by Sigma-Aldrich, St. Louis, MO, USA) at final concentration of 50 nM, transfected with Lipofectamine™ RNAiMAX Reagent (13778150, Thermo Fisher, Life Technologies Waltham, MA, USA), following the manufacturer protocol. Effects of siRNA silencing were tested by individual western blots and qPCR to ensure specificity of the silencing. Scrambled-sequence containing siRNA, nonspecific to any *Rattus norvegicus* gene (Scr siRNA), was used as a control.

### 2.2. qRT-PCR

#### 2.2.1. qRT-PCR Protocol

Total RNA was extracted from cells by the acid guanidinium thiocyanate–phenol–chloroform extraction using Trizol reagent (15596-018; ThermoFisher, Waltham, MA, USA). The reverse transcription was performed using QuantiTect Reverse Transcription Kit ( QiaGen, Venlo, The Netherlands; cat. No. 205310), using 1000 ng of RNA previously quantified by NanoDrop 2000 (Thermo Fisher). Real-time polymerase chain reaction was performed with FastStart Essential DNA Green Master Mix (Roche, Basel, Switzerland). The PCR amplification was carried out with initial denaturation at 95 °C for 20 s, followed by 45 cycles of 95 °C for 3 s and 60 °C for 30 s in a LightCycler 96 (Roche). The primers used were as indicated in Table 1. All of them led to a single amplicon product, when the described qRT-PCR procedure was performed. Data were calculated by the 2^−ΔΔCT^ method (where CT is cycle threshold and ΔΔCT is CT for the gene of interest minus CT of the internal control), having β-actin (“*Actb*”) as an internal control for each experimental condition.

#### 2.2.2. Estimation of mtDNA Copy Number

The mtDNA from model β-cells, INS-1E cells, was isolated by phenol–chloroform extraction. This was followed by SYBR Green qPCR amplification with primers annealing on the *Slco2a1* nuclear gene (encoding solute carrier organic anion transporter family member 2A1) and the *Nd5* mitochondrial gene (bp 11,092 to 11,191 according to GenBank sequences from The National Center for Biotechnology Information, Bethesda, MD, USA) [40]. For primers see Table 1. The ratio between *Nd5* amplicon and half of the nuclear amplicon amounts was taken as the mtDNA copy number per cell.

### 2.3. Protein Separation Methods and Western Blotting

#### 2.3.1. SDS-Electrophoresis and Western Blotting

To determine relative levels of DAPIT/USMG5, ATP synthase α-subunit, PGC-1α and β-actin proteins, cells were lysed in RIPA buffer (Tris-HCl 20 mM, NaCl 150 mM, Na_2_EDTA 1 mM, EGTA 1-mM and NP-40 1%) containing protease inhibitors (1-mg/mL aminocaproic acid, 1-mg/mL benzamidine, 0.2-mg/mL SBTI and 3-mM PMSF) and phosphatase inhibitors (0.012-mg/mL sodium orthovanadate, 4.46-mg/mL sodium pyrophosphate and 4.2-mg/mL sodium fluoride). Total protein concentration was determined by the bicinchoninic acid protein assay (Pierce, Waltham, MA, USA).

Proteins (30 µg) from lysates were separated by electrophoresis in 12% SDS–polyacrylamide gel (SDS-PAGE). Proteins were transferred to a 0.45 µm PVDF membrane, which was blocked with 5% non-fat milk in TTBS containing 0.05% Tween-20 at room temperature. Then, the PVDF membrane was incubated overnight at 4 °C with the primary antibody anti-ATPA (Abcam, Cambridge, MA, USA, ab110273), PGC1 (ThermoFisher, PA5–38021), DAPIT/USMG5 (Abcam, ab108225) or anti-β-actin mAb (Sigma–Aldrich, A2228) at 1:2000 of dilution, followed by incubation with secondary antibody conjugated to peroxidase at 1:10,000 of dilution (Santa Cruz Biotechnology, Dallas, TX, USA) for one hour at room temperature. Immunoreactive bands were visualized using a chemiluminescent reagent (Western Lightning, PerkinElmer, Waltham, MA, USA) according to the procedure described by the manufacturer. Chemiluminescence was detected by the Chemidoc-IT Imaging System (UVP, LLC) and immunoreactive bands were analyzed by densitometry analysis using the ImageJ software (National Institutes of Health).

#### 2.3.2. Isolation of Mitochondria from INS-1E Cells

Mitochondria were isolated by differential centrifugation in ice-cold medium (180-mM KCl, 5-mM MOPS-K buffer, pH 7.2, 2-mM EGTA and 0.5% bovine serum albumin, BSA) according to a published procedure [41]. Final mitochondrial pellets were washed by resuspension/centrifugation in the isolation medium lacking BSA. The protein content was determined by the BCA method (Sigma).

#### 2.3.3. Blue-Native and Clear-Native Electrophoresis

ATP synthase oligomerization was analyzed by one-dimensional blue native (BN-) and clear native (CN-) polyacrylamide gel electrophoresis (PAGE). Protein complexes were extracted from INS-1E cells or INS-1E cell mitochondria using digitonin (Thermo Fisher; 2.5:1, *w:w*, protein:digitonin ratio). The obtained lysates were separated on bis-Tris 3%–13% gels. Following BN/CN PAGE proteins were transferred by wet electroblotting onto PVDF membranes. ATP synthase and DAPIT were immunodetected with monoclonal antibodies against the ATP synthase F_1_α-subunit (Abcam, ab110273) and DAPIT/USMG5 (Abcam, ab108225) after acidic membrane-stripping.

### 2.4. ATP Assay

Before the experiment, siRNA-transfected cells were preincubated for 2 h in the KRH buffer with 0.1% fatty acid-free BSA and indicated glucose concentrations. Quantification of ATP was performed using the ATP Assay bioluminescence kit HSII (Roche). Cells were mixed with boiling lysis buffer (100-mM Tris, 4-mM EDTA, pH 7.75) and further boiled for 2 min. Samples were centrifuged at 10,000× *g* for 1 min. Diluted supernatants were mixed with luciferase reagent and a Synergy HT luminometer was used to read the bioluminescence. To confirm that the assay procedure does not interfere with the ATP concentration determination, internal ATP standards were added to the samples during the initial experiments.

### 2.5. High Resolution Respirometry

Routinely, an oxygraph 2k (Oroboros Instruments GmbH, Innsbruck, Austria) has been used for experiments checking respiration of INS-1E cells as described elsewhere [11,12].

### 2.6. Transmission Electron Microscopy

Cells were cultured on poly-l-lysine–coated petri dishes. For transmission electron microscopy (TEM), cells were fixed for 24 h in 0.1-M cacodylate buffer (pH 7.2) containing 2.5% glutaraldehyde and postfixed in 2% OsO_4_ in the same buffer. For visualization of conspicuous membranes of mitochondria, post fixation was performed with 2% OsO_4_ plus 0.8% K_4_Fe(CN_6_) in a PBS buffer. Fixed samples were dehydrated through an ascending ethanol and acetone series and embedded in araldite –Poly/Bed^®^ 812 mixture (ThermoFisher, Waltham, MA, USA). Thin sections were cut on a Reichert-Jung Ultracut E ultramicrotome and stained using uranyl acetate and lead citrate. Sections were examined and photographed using JEOL JEM-1011 electron microscope. Fine structure measurements were performed using a Veleta camera and iTEM 5.1 software (Olympus Soft Imaging Solution GmbH, Hamburg, Germany).

### 2.7. Experiment with Mice

#### 2.7.1. Mice

C57BL/6 mice were obtained from The Jackson Laboratory, Bar Harbor, MN. Experiments were approved by the Animal Care and Use Committee (Inst. Molecular Genetics, ASCR) in accordance with the European Union Directive 2010/63/EU for animal experiments, U.K. Animals (Scientific Procedures) Act, 1986 and the Guide for the Care and Use of Laboratory Animals (NIH Publication No. 85–23, revised 1996) and the ARRIVE guidelines (www.nc3rs.org.uk/ARRIVE).

#### 2.7.2. Experiments with Hyaluronic Acid Implants

Our protocol was adopted from Praveen et al. [42,43]. Hyaluronic acid implants were a kind gift from Dr. Semira Kwabi from Skin Clinic UK. A Juvederm Ultra 4 hyaluronic acid filler was mixed with d-glucose to a final concentration of 10 M. Such preparation (either 100 μL or 200 μL) was subcutaneously implanted to mice. Typically, two weeks of treatment with hyaluronic acid implants of the designated volumes were performed. Pancreases were removed from three mice and mRNA was isolated using an RNA isolation kit (Qiagen). This was followed by qRT-PCR as described above. Alternatively pancreatic islets were isolated from three pooled pancreases as described elsewhere [40].

### 2.8. Statistical Analysis

Results are presented as mean ± standard deviation (SD) for *N* number of biologic replicates or total number of estimates (*n*). Graphs were plotted and statistical analyses were performed using SigmaPlot 6.0 and SigmaStat 3.1 (Systat Software, San Jose, CA, USA) and ANOVA followed by the Tukey’s test on the pre-validated data; or, alternatively using a Prism (GraphPad Software, San Diego, CA, USA) and an unpaired Student’s *t*-test (when comparing two groups) or one-way nonparametric ANOVA (Tukey’s test) followed by Bonferroni post hoc analysis (for the comparison of more than two groups) or correlation analysis. Statistical significance was set at *** *p* < 0.001; ** *p* < 0.05; * *p* < 0.1.

## 3. Results

### 3.1. Expression of DAPIT and Other F_O_ Subunits Varies according to Metabolic State in INS-1E Cells

INS-1E cells are routinely cultured with 11-mM glucose, thus keeping optimum autocrine factors and *Ins* gene expression [39,41]. Hence, we attempted to study dependencies on glucose for selected subunits of the membrane F_O_ moiety of the ATP synthase, starting with DAPIT. To do this, glucose was first depleted from INS-1E cells, using short prewashing in PBS followed by two 15-min washings in the culturing medium with 3-mM glucose and a half-content of the fetal calf serum. Cells were subsequently incubated at 3 mM, 11-mM and 20-mM glucose in the KRH buffer for up to 120 min. The *Usmg5*/*Dapit* transcript frequently decreased after the two consequent 15 min preincubations with 3-mM glucose (Supplementary File 1; Figure S1a) and for some cell passages we have encountered decreased DAPIT mRNA even after the initial PBS wash. One may regard these conditions as simulating fasting state in vivo, when a slower rate of metabolism is established; nevertheless, for cultured cells this treatment represents a certain form of shock.

Further incubation in KRH containing 11 mM or 20-mM glucose led frequently to restoring the initial levels of DAPIT mRNA already after 30 min (Supplementary File 1; Figure S1a). With further incubations at 20-mM glucose up to 120 min, DAPIT transcript increased up to the ratio to β-actin about 2.5 for some cell passages (Supplementary File 1; Figure S1b). The extent of such elevation was variable, most probably depending on the variability in the initial pretreatment. Nevertheless, specificity of this qRT-PCR assessment was checked in conjunction with pre-transfection by DAPIT siRNA. Indeed, if elevations of DAPIT transcript occurred in control INS-1E^Scrl^ cells (transfected with siRNA containing the scrambled sequence), they vanished when cells were silenced for DAPIT (Supplementary File 1; Figure S1b). Moreover, when cells were left in the medium with 11-mM glucose up to 120 min, DAPIT mRNA started to rise around 60 min (Supplementary File 1; Figure S1c). When glucose was initially set to 20 mM, the elevations of DAPIT mRNA levels were higher around and after 30 min (Supplementary File 1; Figure S1c). We may interpret these elevations as responses to the elevated metabolism and beginning at 60 min even some biogenesis could take place. Investigations of these aspects are reported below.

Dealing with the other three selected subunits of the ATP synthase membrane F_O_ sector, i.e., subunits ***e***, ***f*** and ***g***, their transcripts were less sensitive to pretreatments in the medium containing 3-mM glucose, except of ***g*** (Supplementary File 1; Figure S2a–c). As expected, DAPIT silencing had no effect to the mRNA recovery of these subunits due to the accelerated metabolism (Supplementary File 1; Figure S2d–f). Their recoveries/elevations were nearly synchronous with those found for DAPIT mRNA.

### 3.2. Upregulation of DAPIT in Pancreatic β-Cells in Mice

**Figure 2:**
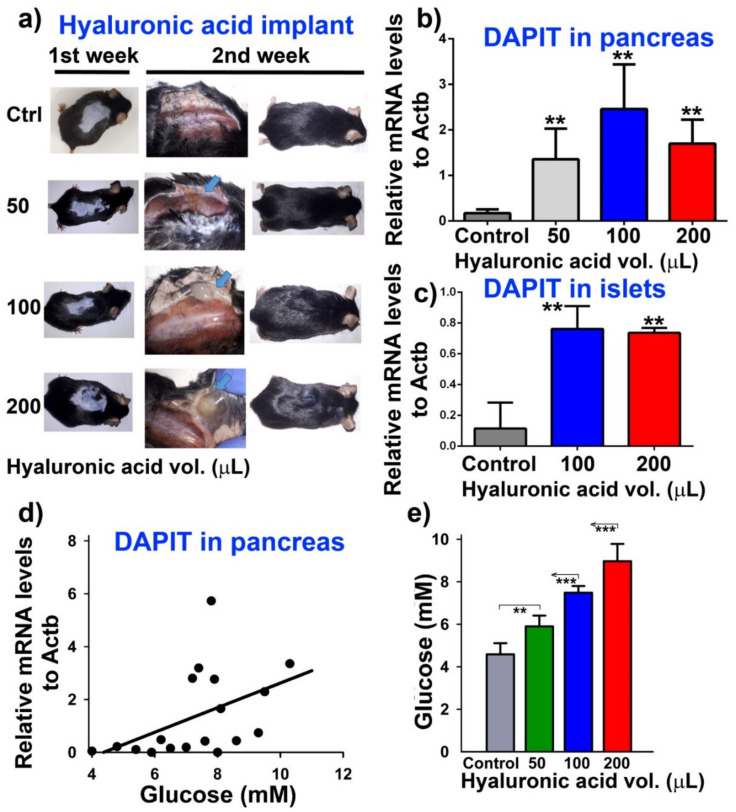
Upregulation of DAPIT in pancreas and pancreatic islets in mice. (**a**) typical experiment, (**b**,**d**) DAPIT mRNA in pancreas (**c**) in isolated pancreatic islets after two weeks of treatment with hyaluronic acid implants of the designated volumes. Data were mutually significantly different (*N* = *n* = 5 mice per group): ** *p* < 0.05 (except 50 vs. 200 µL) as well as data vs. control in (**c**); (**d**) dependence of DAPIT transcript in pancreas vs. glucose; resulting from fasting glucose levels in mice, calibrated relatively to volume of hyaluronic acid (see panel (**e**); *** *p* < 0.05). Spearman coefficient for obtained fit is 0.56; *p* = 0.015.

**Figure 3:**
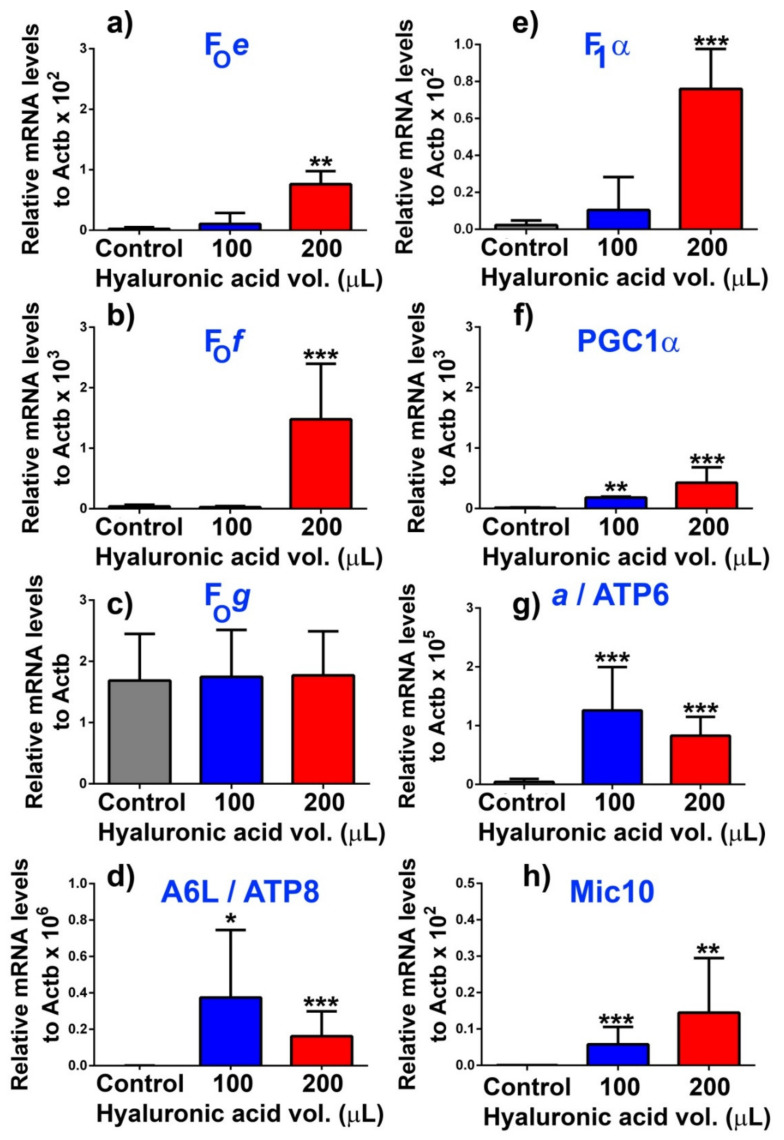
Upregulation of transcripts of other ATP synthase subunits (**a**) F_O_e, (**b**) F_O_f, (**c**) F_O_g, (**d**) A6L/ATP8, (**e**) F_1_α, and (**g**) ATP6 and (**f**) PGC1α and (**h**) and Mic10 in pancreatic islets in mice—after two weeks of treatment with hyaluronic acid implants of the designated volumes. Data except in panel (**c**) are mutually significantly different (*N* = *n* = 5 mice per group): *** *p* < 0.001; ** *p* < 0.05; * *p* < 0.1 (with exception of controls vs. 100 µl in panels (**a**,**b**,**e**).

**Figure 4:**
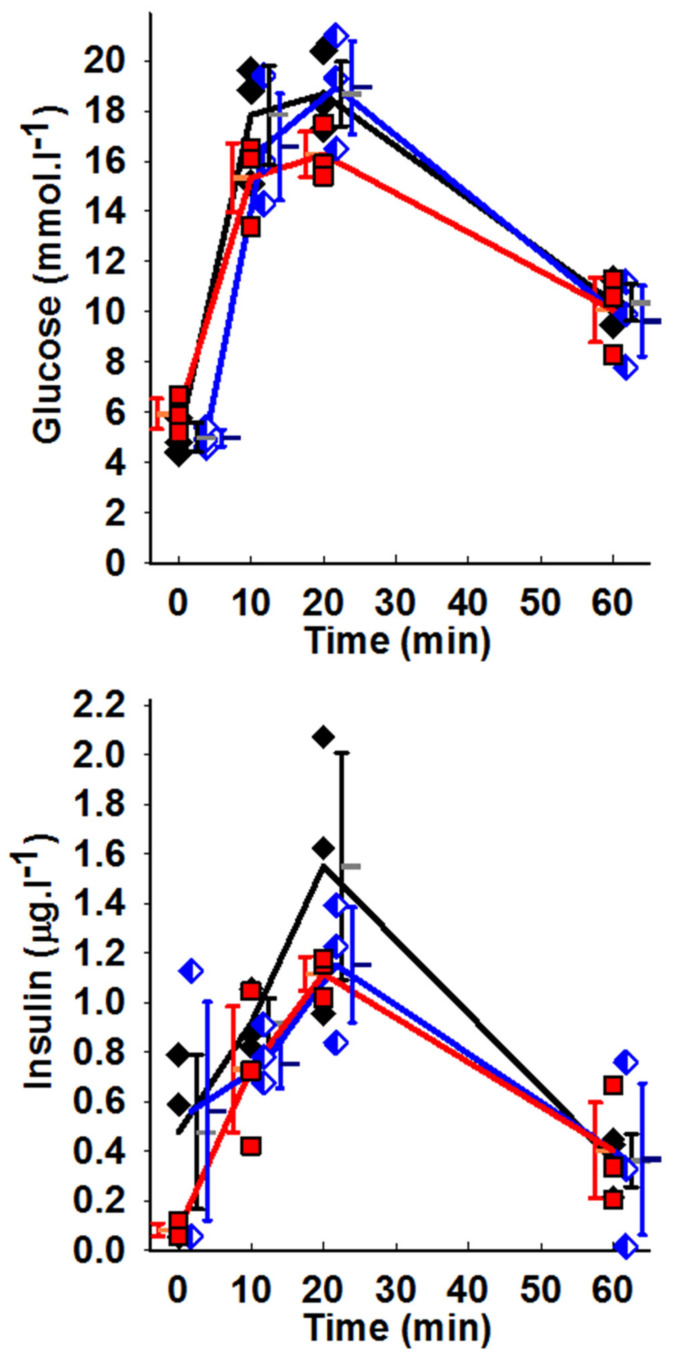
Time course of glycemia and insulin release —i.e., intraperitoneal glucose tolerance test and insulin time course, performed as described in Ref. [8], after a single glucose dose. Mice were treated for two-weeks with hyaluronic-acid implants with no glucose (black symbols represent sham operation); 100 μL (blue symbols) and 200 μL of implants (red symbols). *N* = 3 mice at each time point. Note for rather exhausted fasting insulin levels (at time zero) for treatment with 200 μL of implants.

Next, we investigated, whether any glucose-induced upregulation of DAPIT exists in vivo in mice (Figure 2a–e; Figure 3a–h; Figure 4). The transcript of mouse *Usmg*/*Dapit* was estimated in whole pancreases (Figure 2a,b,d), as well as in the isolated pancreatic islets (Figure 2c). Up to 2 weeks of diffusion of glucose was allowed from the hyaluronic acid implants inserted into the omentum of Black6/J mice (Figure 2a). Attained fasting blood glucose levels reached on average to 7.5 and 9-mM for 100 µL and 200 µL volumes of implants, respectively (Figure 2e). The observed increases relatively to β-actin in whole pancreases (Figure 2b) and isolated islets (Figure 2c) support the existence of the glucose-induced expression of DAPIT in vivo. Similarly, an increase in F_1_ subunit **α** mRNA was pronounced in mice having glucose delivery by the implants (Figure 3e), as well as increases in mRNA of the other selected membrane F_O_ sector ATP synthase subunits (Figure 3a,b,d,f–h), except of subunit ***g*** (Figure 3c). In conclusion, two-week glucose delivery induced expression of the ATP synthase subunits of the membrane F_O_ sector and subunit **α**, as a major subunit of the F_1_ moiety.

Note that the applied procedure did not induce any glucose intolerance as documented by the time courses of insulin release after glucose injection (Figure 4) and concomitantly performed glucose tolerance tests (Figure 4). With higher implant volumes, mice had rather exhausted fasting insulin levels, possibly reflecting starting hypoinsulinemia. Nevertheless, insulin release time course was identical to sham-operated controls (Figure 4).

### 3.3. Glucose Independent but DAPIT-Dependent Mitochondrial Biogenesis in INS-1E Cells

**Figure 5:**
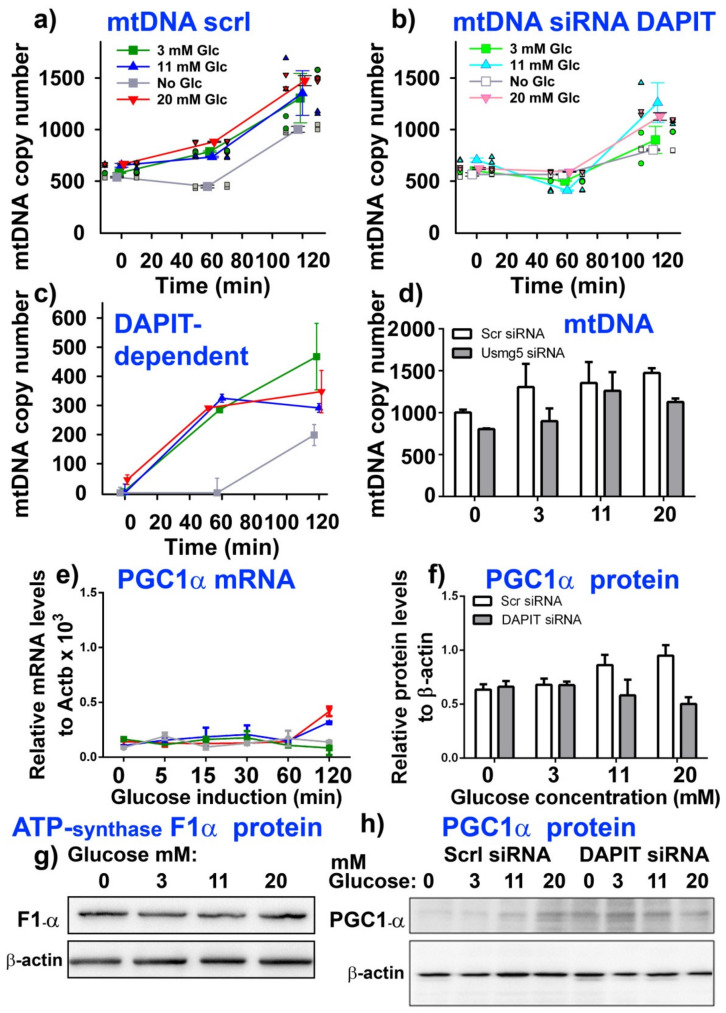
Glucose-induced expression of mitochondrial biogenesis. Time course (**a**–**c**) and glucose concentration dependence (**d**) for DAPIT-dependent increase in mitochondrial DNA copy number. Panels (**a_,_b**) show individual data plus their averages, while (**c**) shows the DAPIT-dependent increase in copy number. Changes in transcript of PGC-1α (**e**) and PGC-1α protein (*N* = 3) (**f**,**h**) and F_1_α protein (**g**) are shown. ANOVA (*N* = 4): (**a**,**b**) vs. zero glucose: ** *p* < 0.05; *** *p* < 0.001. Full size western blot of panel (**g**) and F_1_α mRNA see Supplementary File 1, Figure S4. For (**e**), thresholds yielded by RT-PCR concerning the PGC-1α amplicon were in triplicates as follows: 24.6; 24.4 and 24.5 for samples of zero glucose, whereas samples treated with 20-mM glucose yielded 22.76; 23.06 and 22.9.

Turning attention back to INS-1E cells, we further investigated possible glucose-induced mitochondrial biogenesis, to answer a question whether any glucose-induced replication of mitochondrial DNA (mtDNA) and/or PGC-1α expression exists. Both, when elevated, reflect the initiation of mitochondrial biogenesis. We found that the mtDNA copy number and PGC-1α (mRNA and protein) were not enhanced until 60 min and 120 min, respectively, lasting from the end of pretreatment (from the two 15-min preincubations with 3-mM glucose) (Figure 5a–g). Elevations of mtDNA copy number became significantly ~1.5 higher after 60 min and 3-fold higher after 120 min with all tested glucose concentrations but zero (Figure 5a). For these experiments, INS-1E cells were transfected with siRNA containing the scrambled sequence (i.e., INS-1E^Scrl^ cells were used).

No increase at 60 min, but a smaller increase at 120 min, was recognized for INS-1E cells silenced for DAPIT (Figure 5b; see Supplementary File 1, Figure S3 for silencing efficiency). As a result, only a portion of increases in mtDNA copy number was DAPIT dependent (Figure 5c); however, even this portion did not depend on glucose. Rather permanent mtDNA replication proceeded, as recognized also when no glucose was added while estimating the time course. Thus, the initial effective mtDNA replication was dependent on DAPIT, i.e., on a complete ATP synthase expression/assembly.

PGC-1α mRNA increased apparently only at 120 min (Figure 5e,), similarly to protein levels (Figure 5f,h). In INS-1E silenced for DAPIT, the levels of PGC-1α protein were nearly equal to those in controls for zero and 3-mM glucose but with 20-mM glucose the PGC-1α protein decreased by 25% relative to zero glucose, which was about 50% relative to INS-1E cells transfected with the scrambled siRNA (Figure 5f,h). Levels of PGC-1α were not affected by the pretreatment with 3-mM glucose (not shown). Furthermore, F_1_α subunit mRNA and protein did not change (Figure 5g; Supplementary File 1; Figure S4).

### 3.4. ATP Levels Correlate with DAPIT Expression Induced by Glucose in INS-1E Cells

**Figure 6:**
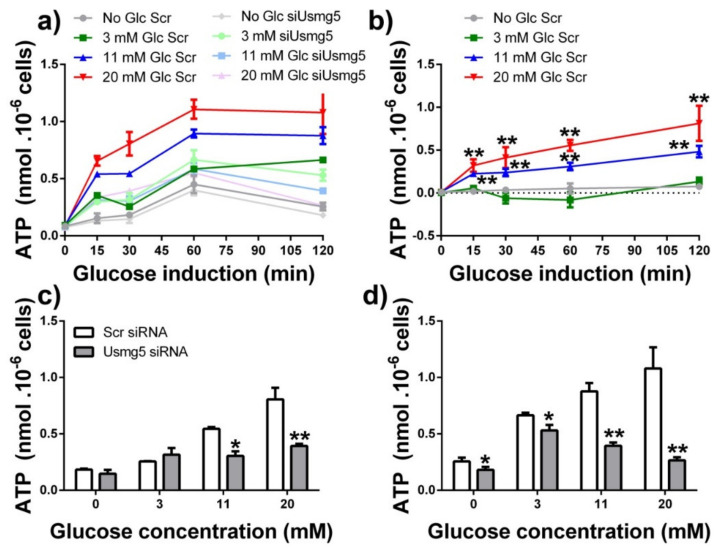
ATP levels in INS-1E cells. (**a**) Time course of the total ATP levels upon glucose induction in INS-1E cells transfected with scrambled siRNA (color coding as in Figure 1) or *Dapit*/*Usmg5* siRNA as indicated; (**b**) DAPIT-dependent part of the total ATP levels (data for *Dapit* siRNA were subtracted from data of scrambled sequence containing siRNA) after glucose induction; (**c**,**d**) Glucose dependencies constructed from the data of panel (**a**). *p* < 0.001 for 11 and 20-mM glucose in (**c**,**d**). Data were significantly different from zero (3 mM) glucose data as indicated (*N* = 3): ** *p* < 0.05; * *p* < 0.1.

Seeking for consequences of DAPIT deficiency, we estimated elevations in the total cellular ATP levels during parallel incubations of INS-1E cells with different glucose concentrations and timing, comparing cells transfected with DAPIT siRNA vs. those transfected with siRNA containing scrambled sequence. Cells were again routinely pretreated by two 15 min pre-incubations with 3-mM glucose. The initial increase was apparent during the 120-min time course for absolute total ATP levels (Figure 6a), despite the constant levels of subunits F_1_α (Figure 5g). Since mitochondrial biogenesis was beginning at around 60 min, as documented above, the initial increases in the total ATP content must originate exclusively from the elevated ATP synthesis by the existing and probably constant number of ATP synthase molecules. This is due to the enhanced metabolism initiated by the added glucose. Such an increase is retarded after 15 min (Figure 6a). Then biogenesis may contribute to the second phase of the observed increase in the total ATP. These two phases are better recognized when the incremental ATP increases were calculated, i.e., when values of the total ATP content was subtracted for samples when no glucose is added (Supplementary File 1; Figure S5).

When induced with 11 and 20-mM glucose, at least half of the incremental ATP increase was dependent on expression of DAPIT, since only about less than half and ~30% of the incremental ATP increase was reached at 30 min and 120 min, respectively, upon DAPIT silencing (Figure 6a). Thus, when induced with 3-mM glucose, the rise of total ATP with time was independent of DAPIT since data were equal to those upon DAPIT silencing (Figure 6a).

Estimating the total ATP content which would be related to the DAPIT presence, we subtracted the time courses measured in INS-1E cells transfected with DAPIT siRNA from controls transfected with siRNA having scrambled sequence (Figure 6b). Clearly, no ATP increase related to DAPIT exists without the induction by glucose and when induced only with 3-mM glucose. In contrast, when induced with 11 and 20-mM glucose, the revealed biphasic DAPIT-dependent accumulation was similar as the net ATP surplus but reaching about 50% of ATP elevations and about 50% initial rate (Figure 6b). If we anticipate that the ablation of DAPIT using siRNA was incomplete, one may expect even higher inhibition of the ATP surplus at completed DAPIT ablation. The glucose concentration dependence at 15 min and 120 min (Figure 6c,d) again indicated a substantial increase in ATP. In DAPIT-deficient INS-1E cells, absolute ATP levels were insignificantly different with 3-mM glucose, but with 11 and 20-mM glucose the total ATP went drastically down. All these results show that the DAPIT, and therefore the complete ATP synthase, is required for the maximum synthesis of ATP.

### 3.5. Phosphorylating Respiration of INS-1E Cells at High Glucose Partially Depends on DAPIT

**Figure 7:**
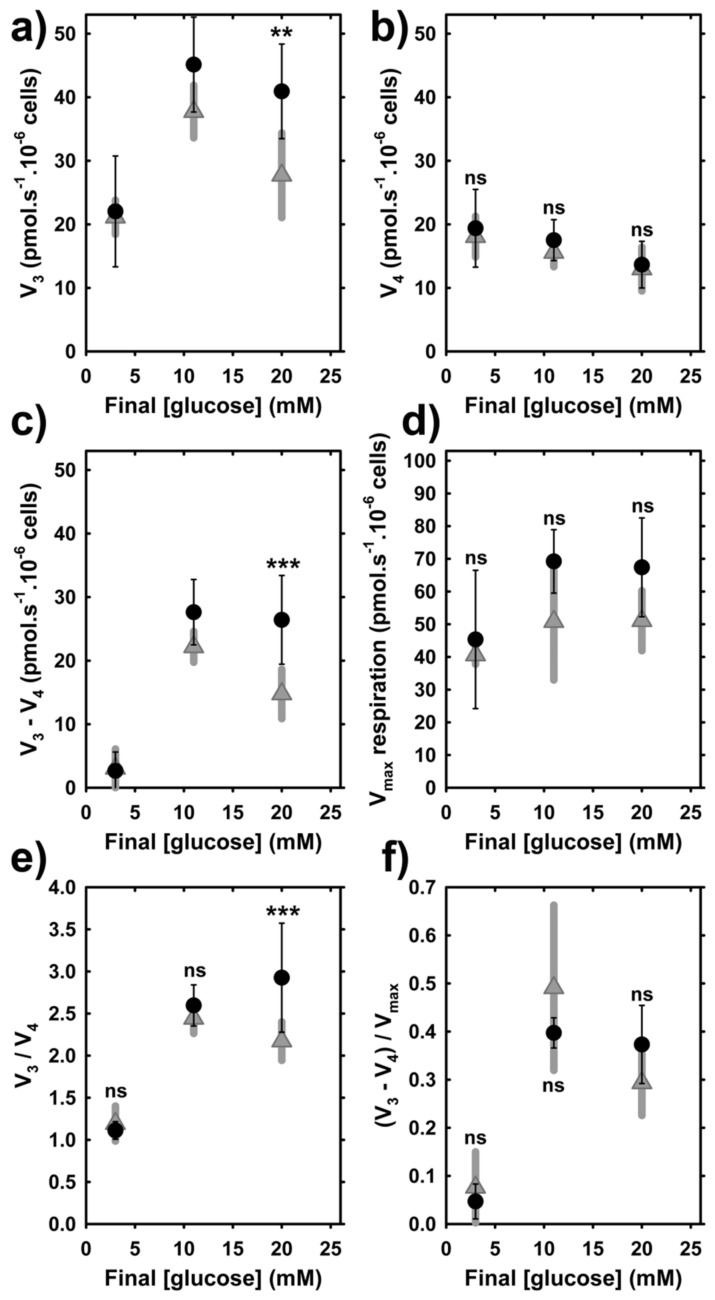
Respiration of control and DAPIT-silenced INS-1E cells. Glucose concentration dependencies for 120 min induction (*N* = 4, *n* = 6–8), after which respiration of was estimated in INS-1E cells transfected with scrambled sequence containing siRNA (*black*) or *Dapit*/*Usmg*5 siRNA (*gray*) (data, SDs) such as: (**a**) phosphorylating (basal) respiration rates (*V*_3_); (**b**) non-phosphorylating respiration rates (*V*_4_); (**c**) ATP synthase-related respiration rates (*V*_3_ − *V*_4_); (**d**) maximum respiration rates *V*_max_ (derived from titration with an uncoupler FCCP); (**e**) Ratios *V*_3_/*V*_4_, i.e., between phosphorylating vs. non-phosphorylating respiration rates; and (**f**) *R*_ATP synthesis_, i.e., portion of total respiratory chain capacity required for the ATP synthesis calculated as (*V*_3_ − *V*_4_)/*V*_max_. *** *p* < 0.001, ** *p* < 0.05 for significant differences of DAPIT-silenced vs. cells transfected with scrambled siRNA.

To estimate independently whether a direct correlation exists between ATP synthesis and DAPIT levels, we evaluated INS-1E cell respiration by standard bioenergetics tests using an oxygraph after 120-min incubations with different glucose concentrations in the KRH buffer, after two previous washings in KRH only (Figure 7a–d). The ratios between phosphorylating vs. non-phosphorylating rates of respiration (*V*_3_/*V*_4_) were derived (Figure 7e). In control INS-1E^Scrl^ cells, these ratios physiologically increased from 1.1 to 2.6 and 2.9, when glucose was raised from 3 mM (representing the insulin non-stimulating levels) to 11 mM and 20 mM, respectively (Figure 7e). The latter two concentrations of glucose are stimulating the release of insulin (see below). The *V*_3_/*V*_4_ ratios with 11-mM glucose upon DAPIT silencing were statistically insignificantly different from those in controls (siRNA with scrambled sequence) but at 20 mM, the *V*_3_/*V*_4_ ratio was significantly lower by ~30%. This perfectly matches the DAPIT contribution to the ATP accumulation (Figure 6a,b).

The total capacity of the respiratory chain can be expressed as the maximum respiration rate *V*_max_, evaluated by titration with an uncoupler FCCP, to reach maximum and saturated respiration. With increasing glucose from 3 to 11 mM, values of *V*_max_ respiration increased 1.5-fold and stayed nearly the same at 20-mM glucose, independently of DAPIT silencing (Figure 7d). We can derive an alternative parameter *R*_ATP synthesis_, expressing the portion of the total respiratory chain capacity required for ATP synthesis, as (*V*_3_ − *V*_4_)/*V*_max_. The calculated values of *R*_ATP synthesis_ reached only 5% at 3-mM glucose for INS-1E^Scrl^ cells or cells transfected with DAPIT siRNA. Independently of DAPIT silencing, the *R*_ATP synthesis_ ratios increased to about 40% with 11-mM and 20-mM glucose (Figure 7f).

### 3.6. Insulin Release Is Insignificantly Affected by the Profoundly Decreased DAPIT Expression in INS-1E Cells

**Figure 8:**
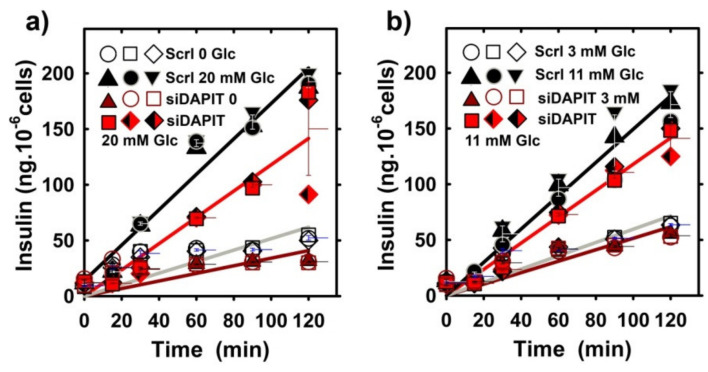
Insulin release in INS-1E^Scrl^ cells vs. DAPIT-silenced cells. Time course of the accumulated insulin with no addition (**a**) and after the addition of 3-mM (**b**), 11-mM (**b**) and 20-mM glucose (**a**). Data originate from *N* = 3 time courses for each condition. Legend: “Scrl“, INS-1E^Scrl^ cells; “siDAPIT”, DAPIT-silenced INS-1E cells.

Next, we estimated time courses of secreted insulin during 120-min incubations of INS-1E cells while using different glucose concentrations added initially (Figure 8a,b). At no glucose addition a small basal rate of insulin release was found (Figure 8a). A similar low linearized rate occurred after the initial addition of 3-mM glucose (Figure 8b). When induced with 11-mM (Figure 8b) and 20-mM glucose (Figure 8a), a nearly linear dependence of the released insulin vs. time up to 120 min reflected the established high rate of insulin secretion at these two stimulating concentrations. Differences between these rates and the basal rate at zero glucose addition represent the net GSIS rate. Surprisingly, the net GSIS rate did not vanish upon DAPIT silencing (Figure 8a,b). When induced with 11 and 20-mM glucose, the net GSIS rate in DAPIT-silenced INS-1E cells accounted for 90% and 80%, respectively, of the mean net GSIS rate for control INS-1E^Scrl^ cells. The effect after around 100 min may contain a contribution of mitochondrial biogenesis occurring during the time course of this experiment, since the PGC-1α transcript 1.5-fold increased during the same period as well as mtDNA copy number.

### 3.7. Morphology of Mitochondrial Cristae Does Not Change with DAPIT Deficiency

**Figure 9:**
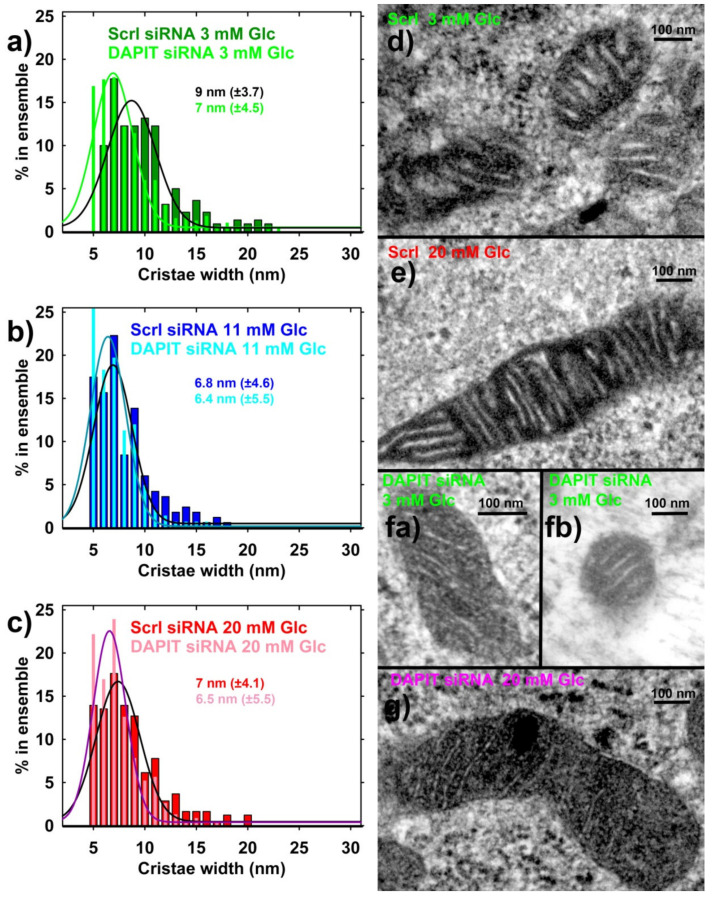
Mitochondrial cristae in DAPIT-deficient INS-1E cells—i.e., silenced for DAPIT, when compared to INS-1E cells transfected with scrambled siRNA (*N* = 2). (**a**–**c**) SDS–polyacrylamide of cristae width; (**d**–**g**) exemplar TEM images of sections of mitochondrial tubules (i.e., “mitochondria”). Independent incubations with the designated glucose concentrations were performed twice, while *n* = 275 crista width estimations were made from 15 TEM images for each condition (*n* = 450 for DAPIT siRNA at 3-mM glucose). Values of most frequent crista width are listed with SDs taken as 0.5 of half-width, as derived from Gaussian fits of the data (displayed on histograms).

Next, we evaluated possible changes in morphology of mitochondrial cristae as consequences of the DAPIT deficiency. We were unable to reach substantial changes in cristae morphology, such as those reported elsewhere [35,36]. The resultant transmission electron microscopy (TEM) images showed that the cristae morphology did not significantly change upon the DAPIT silencing (Figure 9a–g).

**Figure 10:**
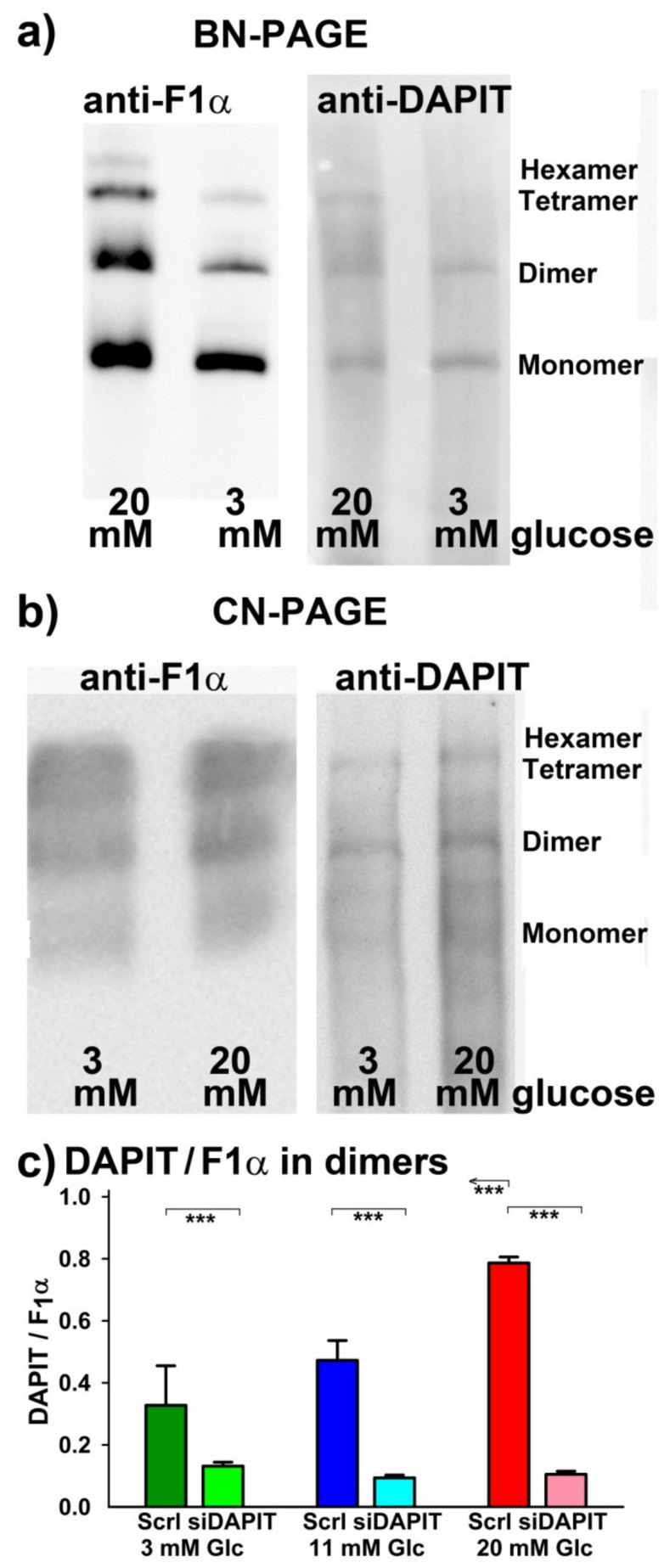
Rough estimations of DAPIT to F_1_α stoichiometry in ATP synthase dimers. (**a**) Exemplar **BN-PAGE** shows missing hexamers and majority of tetramers at 3-mM glucose; (**b**) **CN-PAGE** immunostained with anti-F_1_α or anti-DAPIT antibody as indicated; (**c**) **DAPIT/F_1_α stoichiometry** was estimated from *N* = 3 CN-PAGEs separating ATP synthase monomers/oligomers after dual western blotting. *** *p* < 0.001 (*n* = 3), for significant differences of DAPIT-silenced vs. INS-1E^Scrl^ cells.

However, the previously described [17] cristae narrowing upon transition from a low to high glucose proceeded also in DAPIT-silenced cells, albeit in a lower extent, since cristae width was rather smaller within the estimated ensemble (275, 275 and 450 estimates in 20, 11 and 3-mM glucose, respectively), when compared to INS-1E cells transfected with scrambled siRNA. Further studies are required to investigate what stands behind the literally cell memory regarding glucose levels. Indeed, the in vivo glucose-dependent changes persist during the isolation procedure of mitochondria. The fact that the in vivo situation is reflected by certain persisting structural (e.g., of cristae) and/or conformational pattern suggests that this is due to a specific persisting assembly of the ATP synthase or other cristae-shaping proteins. Note also, that at 3-mM glucose ATP synthase tetramers largely disappeared and hexamers vanished (Figure 10a,b), as reported elsewhere for hypoxic HepG2 cells [18,44].

### 3.8. Rough Estimation of Stoichiometry for the DAPIT Relatively to the ATP synthase F_1_α

Finally, we attempted to derive how the DAPIT to F_1_α stoichiometry may change with the increasing glucose. Moreover, we verified whether such stoichiometry approaches to zero upon DAPIT silencing. Thus, parallel BN-PAGEs (Figure 10a) and parallel CN-PAGEs (Figure 10b) were run, followed by western blotting, and were immunostained either against F_1_α, to distinguish monomeric, dimeric and tetrameric ATP synthase; or against DAPIT (Supplementary File 1; Figure S6), to identify roughly its bound amount to either monomeric, dimeric and tetrameric ATP synthase. Acid stripping of PVDF membranes allowed to cross-correlate antigen amounts.

Typical results of semiquantification from CN-PAGE are shown in Figure 10c, estimating only situation in dimers, i.e., anti-DAPIT antibody staining density ratios relatively to the immunostaining of ATP synthase dimers by anti-F_1_α antibodies. Our attempts to evaluate these ratios relatively to tetramers failed due to a rather highly variable fraction of tetramers occurring in CN-PAGEs. Thus, we repeatedly found that the apparent staining ratio of DAPIT to F_1_α exclusively among the fraction of dimers was increasing at 20-mM glucose (and slightly at 11 mM) relatively to 3-mM glucose (Figure 10c). The expected theoretical maximum saturated stoichiometry should be 2:6 within a single dimer of the ATP synthase. Different antibody efficiency prevents to get absolute stoichiometry values. Note also, that at 3-mM glucose ATP synthase tetramers largely disappeared and hexamers vanished (Figure 10a,b), as reported elsewhere for hypoxic HepG2 cells [18,44].

## 4. Discussion

In this work, we demonstrated that when a fraction predominates of vestigial ATP synthase molecules deficient of USMG5/DAPIT, rat pancreatic β-cells (INS-1E cells) almost do not elevate ATP levels as responding to high glucose. Despite these levels were merely elevated only by 6%, such DAPIT deficiency surprisingly did not inhibit glucose-stimulated insulin secretion (GSIS). The latter result represents a remarkable paradox, specific for pancreatic β-cells. The glucose-stimulated insulin secretion (GSIS) was not substantially hampered in DAPIT-deficient cells. This paradox can be explained on the basis of the recently revisited mechanism for the induction of insulin exocytosis. We demonstrated that H_2_O_2_, originating from NADPH oxidase-4 (NOX4), is essentially required together with ATP, as a logical sum for insulin secretion stimulated with glucose [8]. Candidate NADPH oxidases, i.e., unidentified isoforms, were implied in GSIS previously [45,46,47,48]. Therefore, at virtually no ATP increase, the existing basal ATP levels are sufficient for GSIS, together with NOX4-ensured redox signaling.

Indeed, the basal total ATP in DAPIT-deficient INS-1E cells is sufficient together with elevated H_2_O_2_ (NOX4 was not hampered) to close the ATP-sensitive K^+^ channel and so to trigger insulin granule exocytosis. Apparently, the only negligibly increasing ATP at 11 and 20-mM glucose in DAPIT-deficient INS-1E cells and still existing GSIS provide another independent yet indirect support for the existence of the NOX4-dependent mechanism [8]. Note, that constitutively expressed NOX4 does not need to be assembled [49]; on the contrary the deletion of NOX2, an inducible NOX isoform, enhanced GSIS in pancreatic islets via decrease in superoxide production and elevated cAMP concentrations [48]. Actually, the observation, that despite a very little elevation of ATP in DAPIT-deficient cells, there is still virtually unaffected secretion of insulin responding to glucose, is very similar to the effect of oligomycin described in Ref. [7] (Figure 6D therein).

Our demonstrations that the increased DAPIT expression correlates with the elevated incremental ATP levels is similar to the correlation with elevated ATP previously described in He–La cells [29], which were rather of a weak character. In our case, with a prevailing population of vestigial ATP synthase molecules set by DAPIT silencing [26], we recognized almost no elevation in incremental ATP as a response on higher glucose concentration. This finding supports the requirement of completely assembled ATP synthase for efficient and maximum synthesis of ATP.

It is necessary to further study whether any assembly intermediates of the ATP synthase [50] (or vestigial ATP synthase molecules in deletion experiments, [26]) contribute to the synthesis of ATP at all. Note that our silencing left a substantial amount of DAPIT mRNA expressed. Despite this fact, the surplus accumulation of ATP was decreased by 94%, likewise the extent of increase in phosphorylating/non-phosphorylating respiration ratio, calculated as *V*_3_/*V*_4_ − 1, which decreased by 40%. We also observed similar ATP and respiratory decreases upon silencing of the ATP synthase F_O_ sector subunit ***e*** (Leguina Ruzzi, unpublished).

As we demonstrated in INS-1E cells, expression of the DAPIT and other F_O_ subunits determined the intensity of ATP synthesis. The latter is indicated by the *V*_3_/*V*_4_ ratio, increases with the increasing glucose from insulin non-stimulating (3 mM) to insulin stimulating concentration (>8 mM) [11,12,14,15,16]. However, as we now show, this does not proceed in DAPIT-deficient cells. Hence, a special attention should be paid to the relationships of respiration vs. oxidative phosphorylation in DAPIT-deficient cells. With 20-mM glucose, the *V*_3_/*V*_4_ respiration rate ratio diminished only to ~70% upon DAPIT silencing, whereas the net GSIS rate accounted for 80%. The fraction of respiration employed for the synthesis of ATP was only 5% at 3-mM glucose and was constant at ~40% with 11 and 20-mM glucose. This phenomenon should be independent of the DAPIT-deficiency, since DAPIT does not interact with the respiratory chain supercomplexes.

We also revealed that the two-week delivery of glucose from hyaluronic acid implants elevates transcription of USMG5/DAPIT and other subunits of the membrane F_O_ sector of the ATP synthase; and, as exemplified by the subunit F_1_**α**, one can expect also elevations of other subunits of the F_1_ moiety. Currently, we cannot distinguish whether there was a direct glucose-induction of the selected ATP synthase subunits, whether faster metabolism due to permanently higher glucose presence increased transcription rate; or whether the elevated transcription was evoked by the induced mild hypoinsulinemia in mice. Note that in this way, we simulated chronic states, imposing a rather mild hyperglycemia (7.5 and 9-mM blood glucose) by the constant glucose systemic delivery to mice, using hyaluronic acid implants. One may consider that even if the turnover of DAPIT was faster than the turnover of the other F_O_ or F_1_ subunits, glucose- or metabolism-induced DAPIT expression would ensure the maintenance of complete ATP synthase. Indeed, ubiquitin degradation exists for certain mitochondrial matrix proteins [51] and this may principally differentiate between subunits. In contrast, if autophagy is involved, all subunits would be degraded simultaneously.

In vivo, postprandial and fasting states are alternating [2,5]. For pancreatic β-cells, this means insulin secreting period and basal period. Various secretagogues coming from metabolized meals induce insulin release by different mechanisms during secreting period, while a rather low insulin secretion exists in the basal state [1,2,3,4,5,6]. Traditionally emphasized prominent role of glucose stems not only from the complex mechanisms of GSIS, but is also supported by demonstrations that glucose also serves in the maintenance of expression of *Ins* gene and other β-cell specific genes [4,6,39,52]; or may act in identity self-checking of β-cells [52]. The revealed upregulation of subunits of the ATP synthase membrane F_O_ sector, including USMG5/DAPIT, and upregulation of the subunit F_1_**α**, by two-week glucose delivery is thus only one among numerous other complex processes induced by glucose.

One can also speculate that the glucose/metabolism-induced DAPIT expression may be part of physiological regulations in pancreatic β-cells. This would be enabled, if a fraction of vestigial ATP synthase molecules exists within the ensemble of ATP synthase dimers located in rows along the cristae rims, i.e., the sharp cristae edges. In this case, the newly expressed DAPIT molecules may gradually saturate the vacant sites. These vacant sites may hypothetically arise from a preferable DAPIT degradation. Using DAPIT silencing, we demonstrated that if such degradation existed, it would inhibit ATP synthesis. According this hypothetical view, the preferential rapid expression of DAPIT and its binding into the vacant sites would then switch on ATP synthesis. DAPIT would then act reciprocally to the physiological inhibitor of the ATP synthase, the ATPase inhibitory factor IF1 [11,13].

Speculatively, the DAPIT deficiency (a lower stoichiometry relatively to the F_O_-sector of the ATP synthase) may exist at low glucose (insulin release nonstimulating), such as given by a higher degradation; and this would cause a substantial ATP synthase inhibition. At high glucose (insulin release stimulating), the resulting glucose/metabolism-induced expression of DAPIT could overcome such loss, restoring ATP synthesis. Alternatively, an excessive DAPIT stoichiometry may be established at high glucose, if such an excessive stoichiometry is required to the optimum ATP synthase operation, hence optimum ATP synthesis at its maximum efficiency. We supported these speculations by our results with rough estimations of possible stoichiometry of DAPIT relatively to the rest of the ATP synthase in dimers. One should take these results with caution, since the provided anti-DAPIT antibody may partly cross-react with some other ATP synthase subunits (Supplementary File 1; Figure S6) and thus distort these estimations. However, since DAPIT silencing substantially reduced immunostaining with these anti-DAPIT antibodies, certain specificity for DAPIT is evident. Our findings of a possible variable DAPIT-ATP synthase stoichiometry thus require further testing.

The above speculations may be plausible if degradation of DAPIT or its glucose- or metabolic induction would be independent of the degradation or expression for the remaining subunits of the ATP synthase. The existence of the vestigial ATP synthase molecules in vivo which lack the USMG5/DAPIT protein would be possible at least for a small fraction, since DAPIT is one of the outmost subunits (Figure 1) that is also lastly added among the F_O_-sector subunits of the ATP synthase during biogenesis [26].

A complex assembly of the entire human ATP synthase was described recently to consist of separate steps assembling independently the F_1_-moiety with the c-ring and complex of subunits ***b***, ***e***, ***g*** [50]. Since DAPIT binds the mtDNA-encoded subunit ***a*** [20,26], synchronous processes of translation on ribosomes proximal to mitochondria, adjacent protein import into the mitochondrial matrix and translation by mt ribosomes participate in final steps of the ATP synthase assembly. One may emphasize here that a parallel mtDNA transcription must take place, concerning the mtDNA-encoded subunits ***a***/ATP6 and ATP8/A6 L. When this is impaired, DAPIT cannot bind to the ATP synthase and the resulting vestigial macromolecules should contain even less subunits.

In this respect, we observed that the mtDNA replication is largely independent of glucose- or metabolic induced upregulations. However, the optimum mtDNA replication again requires the completely assembled ATP synthase. This represents a feedback loop. Similar results were obtained for induction of PGC-1α, as one of the factors of mitochondrial biogenesis.

In addition, unlike the published reports for fibroblasts [35,36], we showed the absence of major effects of DAPIT deficiency on morphology of mitochondrial cristae in INS-1E cells. The reason of this discrepancy may stem from more stable mitochondrial network and its rich cristae in INS-1E cells relatively to fibroblasts. That is why we can interpret the above discussed respirometry analyses without a major contribution of cristae morphology changes. As we also demonstrated, DAPIT-deficient vestigial ATP synthase dimers may form thinner cristae. This is supported by our evaluated distribution histograms of cristae width (Figure 9a–c). Nevertheless, unlike in reported observations with mutant DAPIT [35,36], general cristae morphology was not disrupted in DAPIT-deficient INS-1E cells and the previously observed cristae narrowing [17] also took place at high glucose, albeit in a smaller extent. Again, we can explain this by the existing rich cristae in INS-1E cells, when compared to fibroblasts. Hypothetically, DAPIT interactions with the subunit ***g*** may control both ATP synthesis as well as the inner mitochondrial membrane morphology, particularly at the crista rims. This prediction is based on the recently revealed structure of the ATP synthase tetramer, in which interactions contributing to the tetramer formation involve subunits ***g*** from the neighbor dimers with the DAPIT in proximity to each of them [20].

In this way, the release of DAPIT from the dimeric/tetrameric ATP synthase would destabilize rows of ATP synthase dimers since it would disconnect the two neighbor dimers within tetramers. In an opposite, binding of DAPIT into yet vacant sites in the dimeric/tetrameric ATP synthase would strengthen rows of ATP synthase dimers/tetramers. Thus, longitudinal interactions within the rim hypothetically govern the extent or strength of the rim stabilization [18]. A stabilized rim (row of the ATP synthase dimers) may lead to cristae narrowing; and, in contrast, the destabilized rim may enable wide cristae [18,35]. In addition, prolonged INS-1E cell exposure to 3-mM glucose may fragment mitochondrial network [53].

## 5. Conclusions

In conclusion, our results with the DAPIT-deficient rat β-cell line (INS-1E cells) indicate that the vestigial ATP synthase lacking DAPIT exhibits much lower efficiency of ATP synthesis. However, despite the fact that the glucose-induced ATP increment was only around 6% in DAPIT-deficient INS-1E cells, this was still sufficient to initiate GSIS by the mechanism(s) independent of ATP elevation, i.e., independent of the oxidative phosphorylation. The result is compatible with the existence of the recently revealed NOX4-dependent redox signaling essential for GSIS [8]. In general, we demonstrated that the optimum expression of DAPIT is elementary required to reach the maximum ATP synthesis. The elevated expression of DAPIT at high glucose further increases the ATP synthesis efficiency. Moreover, we described rather a compensating response of pancreatic islets cells, i.e., including β-cells, lying in the elevated transcription of the ATP synthase F_O_ subunits ***e***, ***f***, ***g*** and DAPIT, as induced by the enhanced metabolism upon higher glucose intake.

## Figures and Tables

**Table 1:** Primers used to identify individual transcripts.

‘	Protein	Sequence
*Usmg5*	DAPIT	Fw: GATGCCCAATTCCAGTTCAC
		Rev: CCAGGACACATCCATTCTACC
*Atp5i*	subunit F_O_ ***e***	Fw: CACCGTGGCGCCGTATGCCATGCCG
		Rev: AAACCGGCATGGCATACGGCGGCGCACAC
*Atp5j2*	subunit F_O_ ***f***	Fw: GAGAGCTGCCAAGCTGGATA
		Rev: GTCGCCGTTCGTGTTTGAG
*Atp5I*	subunit F_O_ ***g***	Fw: CACCGCATCCGTAACCTCGCGGACA
		Rev: AAACTGTCCGCGAGGTTACGGATGC
*Atp6*	subunit F_O_ ***a***	Fw: CGCCACCCTAGCAATATCAA
		Rev: TTAAGGCGACAGCGATTTCT
*Minos1*	Mic10	Fw: GAACATCCCAGCGGGAGAAA
		Rev: ACTGATGGCACTGTCACAGGA
*Atp5f1a*	subunit F_1_ **α**	Fw: TCCAAGCAGGCT GTTGCTTAC
		Rev: TGT AGGCGGACACATCACCA
*Pgc1a*	PGC-1**α**	Fw: GGAGTGACATAGAGTGTGCTG
		Rev: CGCGGGCT ATT GTTGTACT
*Actb*	Actin	Fw: ATCTGGCACCACACCTTC
		Rev: AGCCAGGTCCAGACGCA
*Nd5* of mtDNA	ND5	Fw: AACTCCCGTCTCTGCCCTAC
		Rev: GGCCTAGTTGGCTGGATGTT
*Slco2a1*	SLCO2A1	Fw: GCAAACTGGGTCATTGCCT
		Rev: CCCTCCAAGAGCCGTTTTCC

## Supplementary Materials

The following are available online at https://www.mdpi.com/2218-273X/10/7/1026/s1, Figure S1: Typical changes in DAPIT mRNA levels in INS-1E cells; Figure S2: Downregulation/recovery of transcripts for F_O_ subunits ***e***, ***f*** and ***g***; Figure S3: Typical results of DAPIT silencing; Figure S4: western blots of F1α; Figure S5: Surplus ATP accumulation; Figure S6: Anti-DAPIT/USMG5 antibodies recognize a complex above ~75 kDa.
