# Supplementary material for: Glucose-Induced Expression of DAPIT in Pancreatic β-Cells
Source: Biomolecules. 2020 Jul 10;10(7):1026. doi: 10.3390/biom10071026 (PMC7408392; doi:10.3390/biom10071026)

SUPPLEMENTARY DATA

Figure S1

Typical changes in DAPIT mRNA levels in INS-1E cells: a) upon decreasing glucose down from 11 mM; or **b)** their recovery upon restoration of glucose to 11 mM or to a surplus 20 mM glucose; or **c)** for routinely cultured cells (N=2) transferred to medium containing 20 mM glucose. Panels **a)** and **b)** show separate experiments: **a)** DAPIT to β-actin mRNA ratios for ascribed conditions; ANOVA (n=3): (** P<0.05; * P<0.1; ns, non-significant). **b)** Time courses for the DAPIT to β-actin mRNA ratios evolved after the settings to the indicated concentrations of glucose in INS-1E^{Scrl} cells pretreated in media with 3 mM glucose (time zero point should correspond to the green bar in panel **a**). Data were calculated by the $2^{-\Delta\Delta CT}$ method (see 2.2.1.) at each time point (n=3) when induced by 3 mM (green), 11 mM (blue) and 20 mM glucose (red) or not-induced ("zero", gray; black for DAPIT-silenced cells). Light colors (light green, aquamarine and pink) show the data points obtained in DAPIT silenced cells (at 3 mM, 11 mM and 20 mM glucose, respectively). ANOVA: Significances related to zero glucose are denoted by asterix (*** P<0.001; ** P<0.05), while those related to the initial (zero) time point are denoted by hashtags ($^{\#\#\#}$ P<0.001).

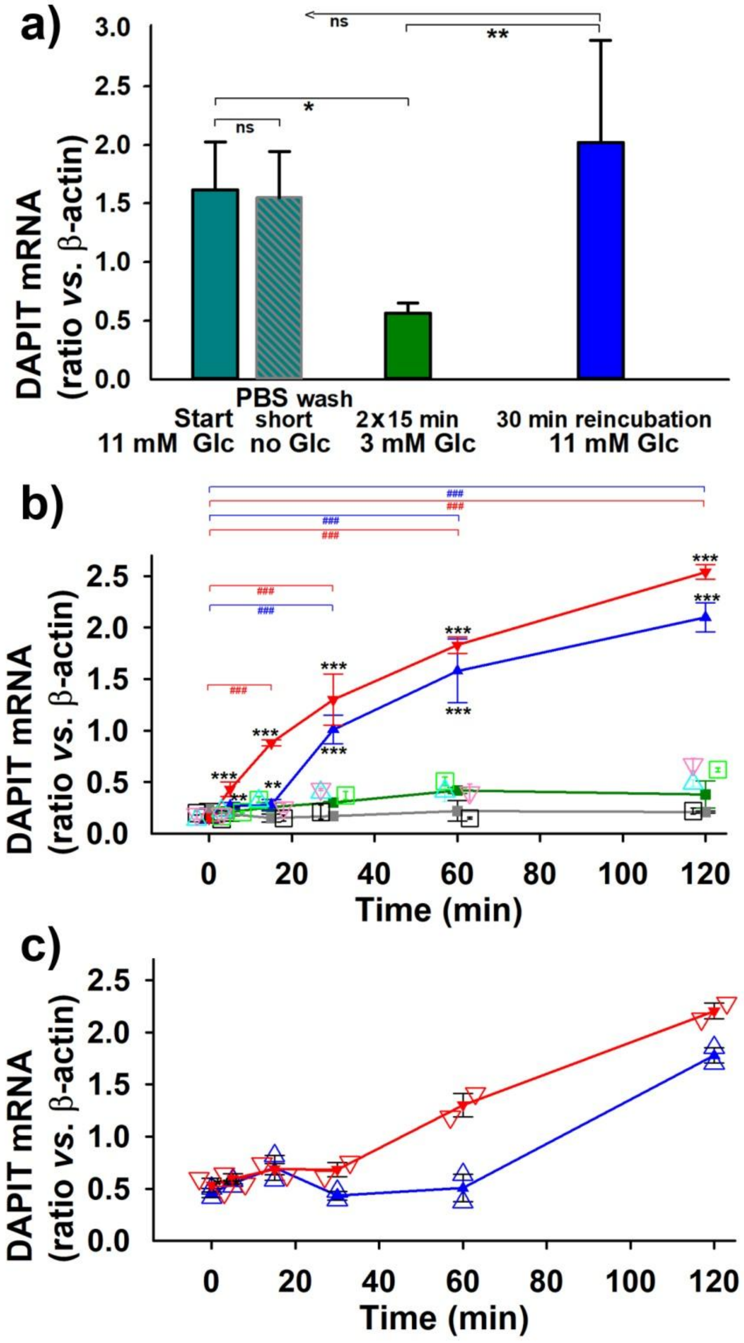

Figure S2

Downregulation/recovery of transcripts for ATP-synthase F_O subunits *e*, *f*, and *g* – encountered mRNA down-regulations at slow metabolism for selected F_O subunits (**a–c)** as induced by the standard pretreatment in media containing 3 mM glucose (see Methods) and their recoveries. ANOVA (n=3): (*** P<0.001; ** P<0.05;* P<0.1; ns, non-significant). Arbitrary units are used.
d– f) Typical changes in mRNA levels relatively to β-actin mRNA of selected F_O subunits upon restoration of glucose from 3 mM (time zero) to 11 mM or to a surplus 20 mM. Time courses evolved after the settings to the indicated concentrations of glucose in INS-1E^{Scrl} cells pretreated in media with 3 mM glucose (Note the point of time zero should correspond to the green bars in panels a–c). Data were calculated by the $2^{-\Delta\Delta CT}$ method (see 2.2.1.) at each time point (n=3) when induced by 3 mM (green), 11 mM (blue) and 20 mM glucose (red) or not-induced ("zero", gray; black for DAPIT-silenced cells). Light colors (light green, aquamarine and pink) show the data points obtained in DAPIT silenced cells (at 3 mM, 11 mM and 20 mM glucose, respectively). ANOVA: Significances related to zero glucose are denoted by asterix (*** P<0.001;** P<0.05), while those related to the initial time point are denoted by hashtags (### P<0.001).

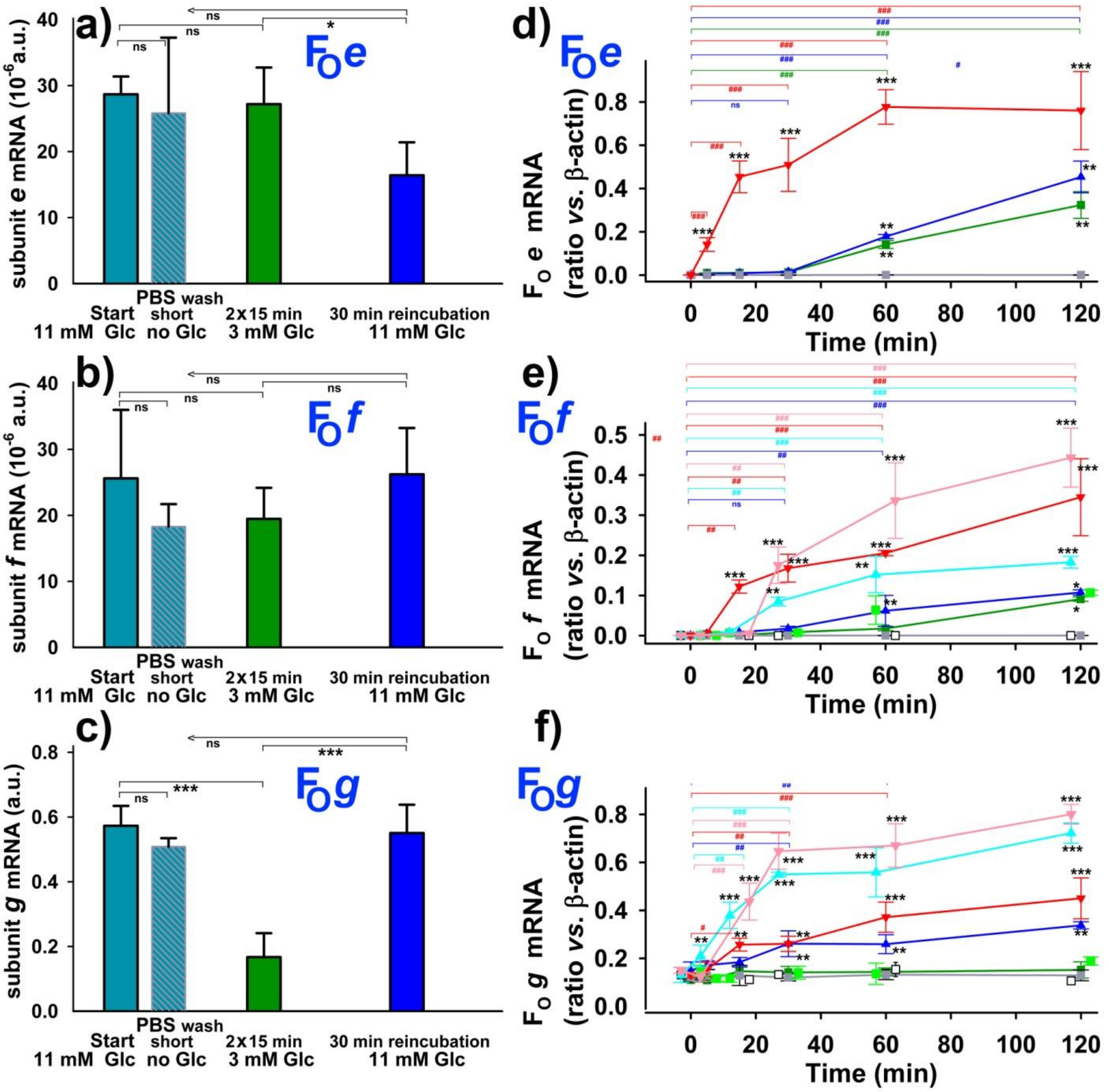

Figure S3 Supplement to Figure 5a–f and 5h

Typical results of DAPIT silencing are shown in **Fig.S3a** for DAPIT/USMG5 mRNA in INS-1E cells transfected with

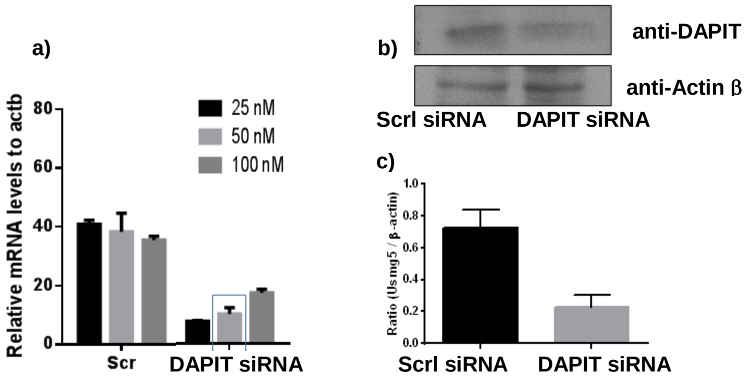

siRNA of scrambled sequence (“Scrl”) and with DAPIT siRNA; panel **b**) illustrates typical Western blots immunostained by reportedly anti-DAPIT antibodies, while panel **c**) presents quantification of *N*=5 experiments.

Figure S4 Supplement to Figure 5g

Exemplar Western blots out of N=3 biological replicates are shown for subunit $F_1\alpha$ (**a**) or β-actin (**b**) concerning data of Figure 5g. Moreover, $F_1\alpha$ mRNA (*dashed bars*) and protein levels (*black bars*) are shown for the alternative 2hr-incubations without the previous pretreatment in 3 mM glucose (**c**). Thus the cells were taken from the maternal cell passage and were incubated for 2 hrs in the culturing medium with normal fetal calf serum content, but without pyruvate containing the indicated glucose concentrations.

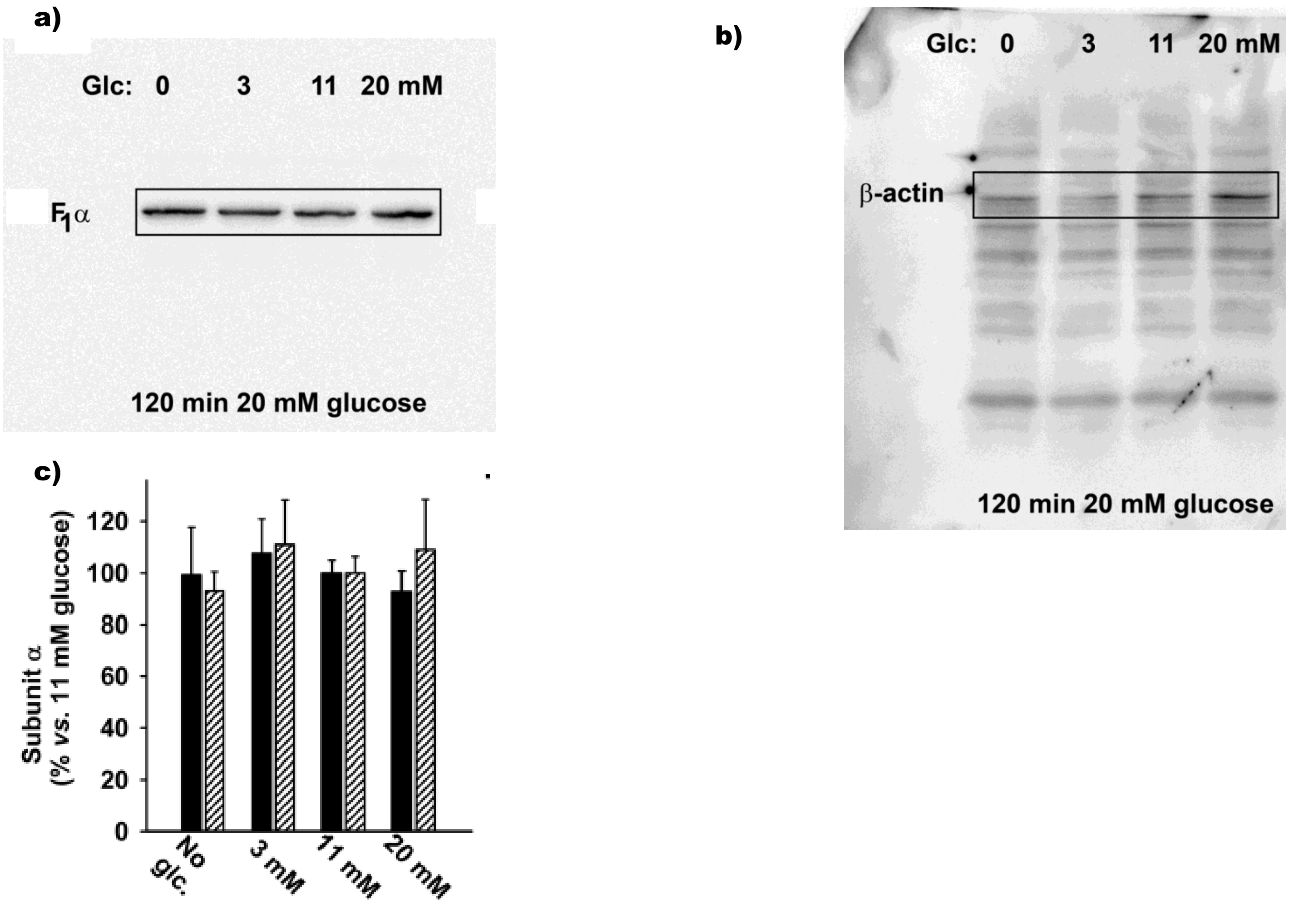

Figure S5 Supplement to Figure 6

Fig.S5 Surplus ATP accumulation as induced with indicated glucose doses for INS-1E cells transfected with scrambled siRNA (shades of blue) and with DAPIT siRNA (shades of magenta and pink). Values represent the net ATP elevations, *i.e*. values with subtracted background.

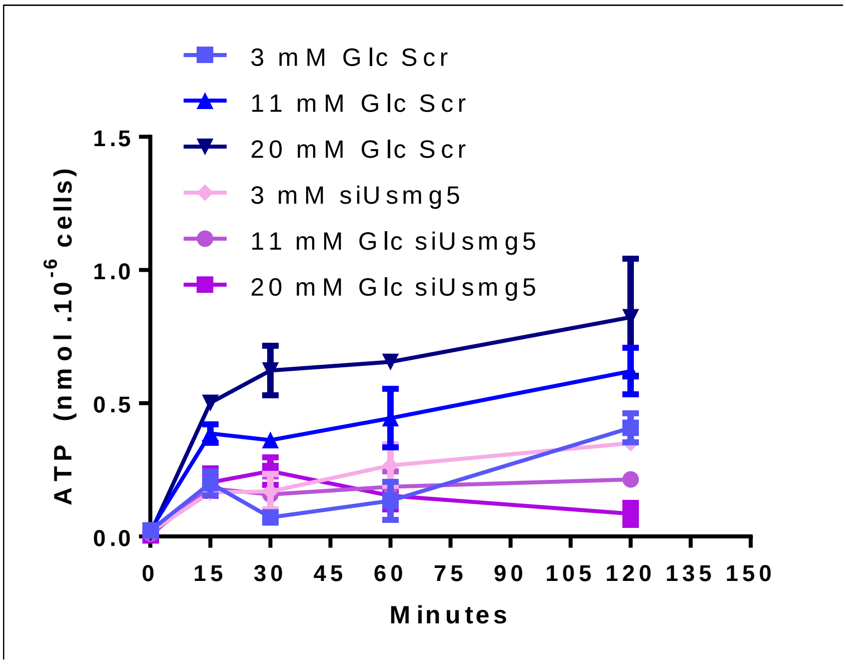

Fig.S6 Supplement to Figure 10

***Anti*-DAPIT/USMG5 antibodies recognize a complex above ~75 kDa**
Full-scale western blots are shown for DAPIT (**a**) and actin β (**b**), "actin b") from an exemplar SDS-PAGE gel. Similar Western blot was made also against the samples of INS1E cells silenced for DAPIT as shown in the panel **c**), including the DAPIT protein semi-quantification.

a)

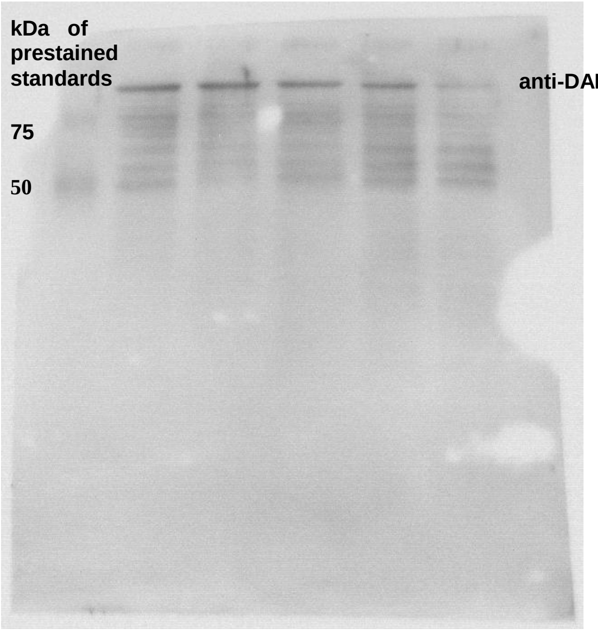

b)

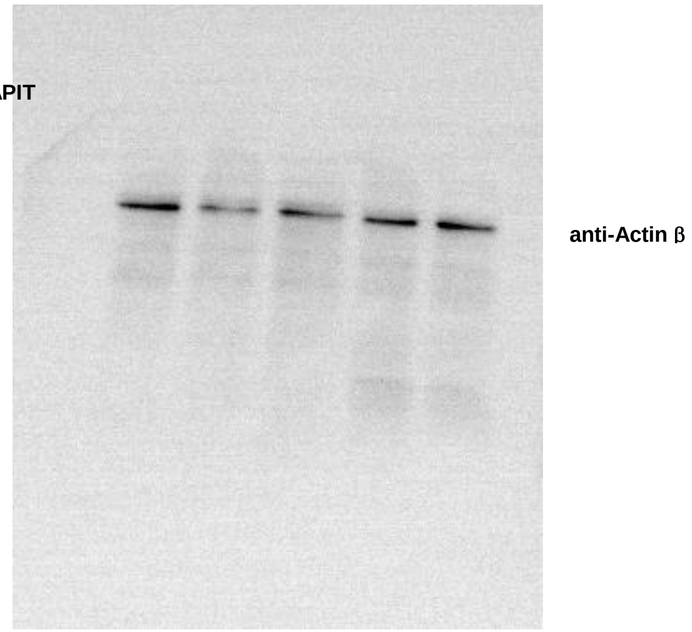

Supplement: Supplementary file 1 [file biomolecules-10-01026-s001.pdf]
